# Net albumin leakage in patients in the ICU with suspected sepsis. A prospective analysis using mass balance calculations

**DOI:** 10.1186/s13054-025-05323-9

**Published:** 2025-03-08

**Authors:** Dag Seldén, Nicolas Tardif, Jan Wernerman, Olav Rooyackers, Åke Norberg

**Affiliations:** 1 Perioperative Medicine and Intensive Care, B31, Karolinska University Hospital, Huddinge, Sweden; 2 Department of Clinical Science Intervention and Technology (CLINTEC), Karolinska Institutet, Hälsovägen, 141 86 Stockholm, Sweden

## Abstract

**Introduction:**

Albumin kinetics in septic shock have been extensively studied, but clinical recommendations remain weak. An increased transcapillary escape rate (TER) of albumin has been demonstrated, though TER does not account for lymphatic return. Mass balance calculations, considering lymphatic return, have been used to assess net albumin leakage (NAL) in major surgery but not in sepsis.

**Objectives:**

This study aimed to evaluate NAL in ten ICU patients with suspected sepsis, hypothesizing a net positive leakage. Secondary aims included investigating associations between NAL and fluid overload, glycocalyx shedding products, and cytokines, as well as identifying factors associated with it.

**Methods:**

This prospective, observational study included ten patients within twelve hours of ICU admission for suspected sepsis at Karolinska University Hospital Huddinge. Albumin, hematocrit, and hemoglobin levels were sampled at 0, 1, 2, 4, 8, and 24 h. NAL was estimated using mass balance calculations, comparing proportional changes in albumin and hemoglobin concentrations over time, adjusted for albumin and hemoglobin infusions and losses. A proportionally greater decrease or smaller increase in albumin compared to hemoglobin indicated NAL, representing the net leakage from the circulation to the interstitium minus lymphatic return.

**Results:**

Over 24 h, patients exhibited a net positive albumin leakage to the interstitium of 8 ± 10 g (*p* = 0.029). NAL showed no correlation with glycocalyx shedding products or fluid overload but had a weak correlation with interleukin-6 and interleukin-8 in the first 4 h. Albumin infusions appeared to increase net leakage.

**Conclusion:**

This study demonstrated a net positive albumin leakage of 8 ± 10 g over 24 h in ICU patients with suspected sepsis, with a weak early correlation to pro-inflammatory cytokines but no significant link to fluid balance or glycocalyx shedding. Notably, albumin infusions were associated with increased net leakage.

**Supplementary Information:**

The online version contains supplementary material available at 10.1186/s13054-025-05323-9.

## Background

Sepsis is a leading cause of mortality worldwide [1]. Intravenous crystalloid infusion is a cornerstone in the treatment of sepsis to maintain organ perfusion and is the recommended first line therapy [2]. Fluid overload is related to poor outcome [3, 4], however, a restrictive fluid therapy has not shown any improvement in outcome [5, 6]. Human albumin solution has a large molecular size and a negative charge which could help retain fluid intravascularly and prevent it from leaking out to the interstitium. Yet, the volume sparing effect of albumin infusion in sepsis is limited and whether it leads to improved outcomes is still under debate [7, 8]. Therefore guideline recommendations for albumin infusions in sepsis are weak [2]. Still, albumin is commonly used throughout the world [9] and therefore further research into the physiology of albumin and the effects of albumin supplementation on this is warranted.

In a paper by Fleck et al. [10], increased transcapillary escape rate (TER) of albumin from the vascular system in septic patients was detected. However, TER only describes the loss of albumin and not the lymphatic return from the interstitium to the circulation. Recently, the net albumin leakage (NAL), i.e. the leakage from the circulation minus the lymphatic return has, been assessed in surgical patients using mass balance equations, suggesting an accumulation of albumin in the interstitium [11–13]. Both cumulative perioperative albumin shift and capillary leakage rate are terms that have been used to describe this net leakage.

Capillary patency, as measured by TER, can be influenced by several factors, including transmural pressure [14, 15] and plasma volume expansion [16]. Damage to the endothelial glycocalyx, a vital component of vascular integrity, represents another mechanism and can occur due to inflammation or possibly rapid fluid administration [17]. Glycocalyx shedding products can be detected in the circulation as markers of endothelial injury [18]. Elevated levels of these shedding products have been reported in conditions such as sepsis and after major abdominal surgery, where they have been shown to correlate with cytokine levels [19]. However, a clear correlation with mortality has yet to be shown [20].

Until now the net leakage of albumin has not been evaluated in patients with sepsis nor has it been correlated to the total fluid overload, markers of inflammation and glycocalyx damage.

The primary aim of this pilot study was to assess NAL in patients with suspected sepsis, early during their ICU stay. Secondary aims were to describe kinetics of glycocalyx shedding products, cytokines and fluid overload and to evaluate its potential correlation to NAL. Finally, if possible, factors contributing to NAL would be investigated. We hypothesize that NAL will be positive and to be correlated to glycocalyx shedding, inflammation and fluid overload.

## Methods

This prospective observational pilot study was conducted in the ICU at Karolinska University Hospital in Huddinge, a tertiary referral hospital. We aimed to include 10 patients treated for sepsis in the ICU with frequent analyses during the first 24 h of their treatment. Inclusion criteria were patients aged 18 years or older with suspected sepsis who received antibiotics and could be recruited within 12 h of ICU admission. Exclusion criteria were absence of consent or lack of research staff. Data collection took place between May and July 2017. Patient characteristics are presented in Table 1.

**Table 1:** Patient characteristics (n = 10) with sepsis on admission to the ICU

	Patients *n* = 10
Length (m) (mean, ± SD)	1.79 ± 0.1
BMI (kg/m^2^) (median, range)^1^	23 (17–33)
Sex (male) (%)	100
Age (years) (median, range)	60 (53–85)
Noradrenaline dose (µg/kg/min) (median, range)	0.017 (0–0.30)
Lactate (mmol/L) (median, range)	2 (1–12)
Plasma albumin (g/L) (mean, SD)	20 ± 5
Hemoglobin (g/L) (median, range)	72 (88–149)
CRP (mg/L) (mean, SD)^2^	213 ± 114
Hematologic or metastatic cancer (%)	50
Liver cirrhosis (%)	10
SOFA score (mean, SD)	9 ± 5
SAPS III (mean, SD)	75 ± 18
Antibiotic treatment during study period (%)	100
Septic shock (%)^3^	80
Delta weight gain during study period (kg) (mean, SD)	3.5 ± 4.4
Invasive or noninvasive mechanical ventilation during study period (%)	50

Values are presented as mean ± standard deviation, median (range) or percentages as appropriate. ^1^BMI = body mass index, ^2^CRP = C-reactive protein, ^3^Septic shock defined as patient with suspicion of sepsis, treated with antibiotics, vasopressors and with a lactate ≥ 2 at the same time

### Mass balance calculations

**Fig. 1:**
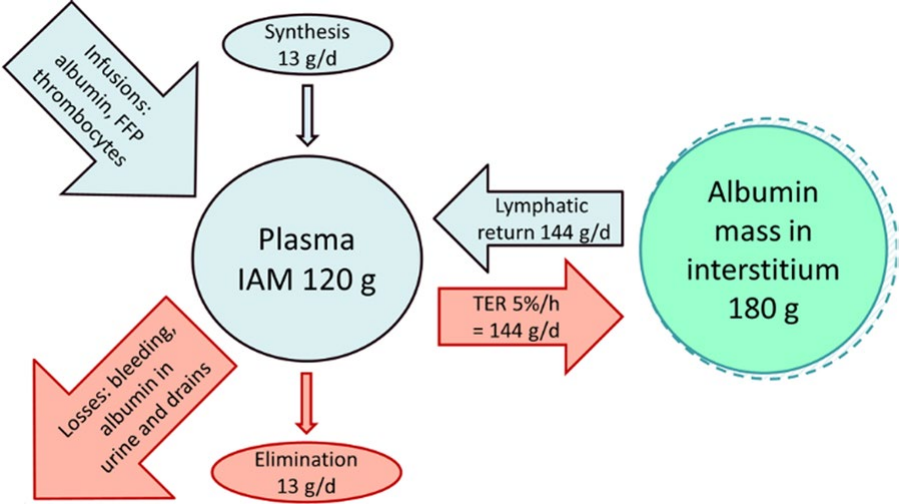
Albumin turnover and metabolism in a healthy adult. This overview illustrates albumin turnover between the intravascular albumin mass (IAM = 120 g) and the interstitial pool (180 g). Transcapillary escape rate (TER = 144 g/day) is balanced by lymphatic return (144 g/day). Synthesis (13 g/day), elimination (13 g/day), infusions (e.g., albumin, platelets, FFP), and losses (e.g., bleeding, urinary albumin, drains) dynamically influence IAM. Synthesis and elimination are not included in the mass balance calculations but are unlikely to affect results over the 24-h study period. IAM = Intravascular Albumin Mass; MHb = Intravascular Hemoglobin mass; FFP = Fresh Frozen Plasma

Albumin mass balance calculations have been previously described [11]; see the supplement for the full calculations and Fig. 1 for an overview of albumin turnover. In short, it assumes that hemoglobin, as opposed to albumin, does not leave the bloodstream through capillary leakage. The NAL from the circulation, supposedly to the interstitium, is then calculated in steps. First, Nadler formula estimates a baseline blood volume using anthropometric data [21]. The plasma volume can then be estimated multiplying the blood volume with 1 minus the measured hematocrit. Thereafter repeated and simultaneous sampling of plasma albumin, hemoglobin, and hematocrit while accounting for losses and infusions of albumin and hemoglobin is conducted. Finally, delta albumin is compared with delta hemoglobin. If albumin concentration is decreased proportionally more or raised proportionally less than hemoglobin over time, this is interpreted as a NAL, assumingly to the interstitium. The opposite situation is interpreted as albumin net recruitment to the circulation, via lymphatic flow. Bleeding is corrected for and should have a negligible effect on NAL. Plasma volume changes are accounted for as the result of changes in hematocrit. Synthesis and elimination of albumin are not included in the mass balance calculations but are unlikely to affect results over the 24-h study period.

### Sampling and analyses

Blood sampling for albumin concentrations, hemoglobin and hematocrit was performed at hour 0, i.e., time of inclusion, and then at hour 1, 2, 4, 8, 24. Albumin samples were taken in tubes with EDTA spun at room temperature at 2000 g, and plasma was then stored at − 20 °C until analyses for albumin by nephelometry (IMMAGE® 800; Beckman Coulter AB, Bromma, Sweden) at the Division of Clinical Chemistry, Karolinska University Hospital. Hemoglobin and hematocrit were measured using a blood gas analyzer (ABL800 FLEX; Radiometer Medical ApS, Brønshøj, Denmark). Albumin and hemoglobin concentrations were analyzed from drains and urinary catheters that were already in situ, but no new catheters or drains were placed solely for this study. Albumin concentrations in drain samples were analyzed using an automated analyzer (Indiko, Thermo Fisher Scientific, Stockholm, Sweden). Concentration of albumin in fresh frozen plasma and thrombocytes as well as concentration of hemoglobin in red blood cell transfusions were based on data from Norberg 2016 [11]. Samples for markers of inflammation and glycocalyx shedding components were taken at timepoints 0, 4 and 24 h in tubes with EDTA, spun at 4 °C 2000 g, and stored at -80 °C until analysis.

The glycocalyx components syndecan-1 (Human CD138 ELISA kit, Diaclone, # 950.640.096) and hyaluronan (Hyaluronan Immunoassay, Quantikine ELISA, R&D Systems, # DHYAL0) were chosen as markers of endothelial injury. Eight different cytokines (Bio-Plex Pro Human Cytokine 8-plex, BioRad, # M50000007A) were analyzed, interleukin-2, interleukin-4, interleukin-6 (IL-6), interleukin-8 (IL-8), interleukin-10 (IL-10), granulocyte–macrophage colony-simulation factor, interferon-γ, tumor necrosis factor-α (TNF- α). Only IL-6, IL-8, and TNF- α showed values above detection limit in most patients and were included in the results. Reference ranges were based on analysis of samples from 35 healthy individuals participating in another study in which our group was involved [22]. All samples were analyzed by ELISA in accordance with the manufacturer’s instructions.

### Statistics

Data are presented as mean ± SD or median (range), as determined by the Shapiro–Wilk test of normality. The one-sample t-test was used to assess if NAL differed significantly from zero. To compare two time points, the dependent t-test was applied. Due to missing data (3%) and skewed distributions, cytokine and glycocalyx shedding product concentrations were first log-transformed and then analyzed using either a mixed-effects model or repeated measures ANOVA with the Greenhouse–Geisser correction, which does not assume sphericity [23]. Associations between NAL, glycocalyx shedding products, and cytokines were assessed using area under the curve values analyzed in a correlation matrix (Pearson’s coefficient). Given the high resolution of our data and the dynamic state assumed in the early part of the patient’s disease state, correlations were performed for the periods 0–4 h and 4–24 h. In a post hoc analysis, Spearman’s rank correlation coefficient was used to evaluate if the albumin levels at the time of inclusion was associated with the NAL and to determine if the amount of albumin administered influenced NAL. Finally, simple linear regression was used to analyze the delta change in administered albumin versus NAL.

As NAL has not previously been evaluated in septic patients, we based our sample size estimation on the results from Norberg [11], where a NAL of 24 ± 17 g corresponding to an effect size of 1.4 (difference divided by standard deviation) was found. To detect a significant difference from zero with a power of 0.9, inclusion of at least 8 patients was necessary. Considering the large heterogeneity of ICU patients, we aimed to include 10 fully evaluable patients.

### Ethics

The study was approved by the Swedish Ethical Review Authority (EPN 2016/2064) in Stockholm and performed in accordance with good clinical practice. Patients was screened and included with a presumed consent, allowing blood sampling to a maximal volume of 20 ml. As soon as possible the patient or next of kin was approached by the study team with written and oral information to obtain delayed consent. In the absence of consent collected samples were destroyed, however none of the eligible patients declined participation.

## Results

### Mass balance calculations

**Fig. 2:**
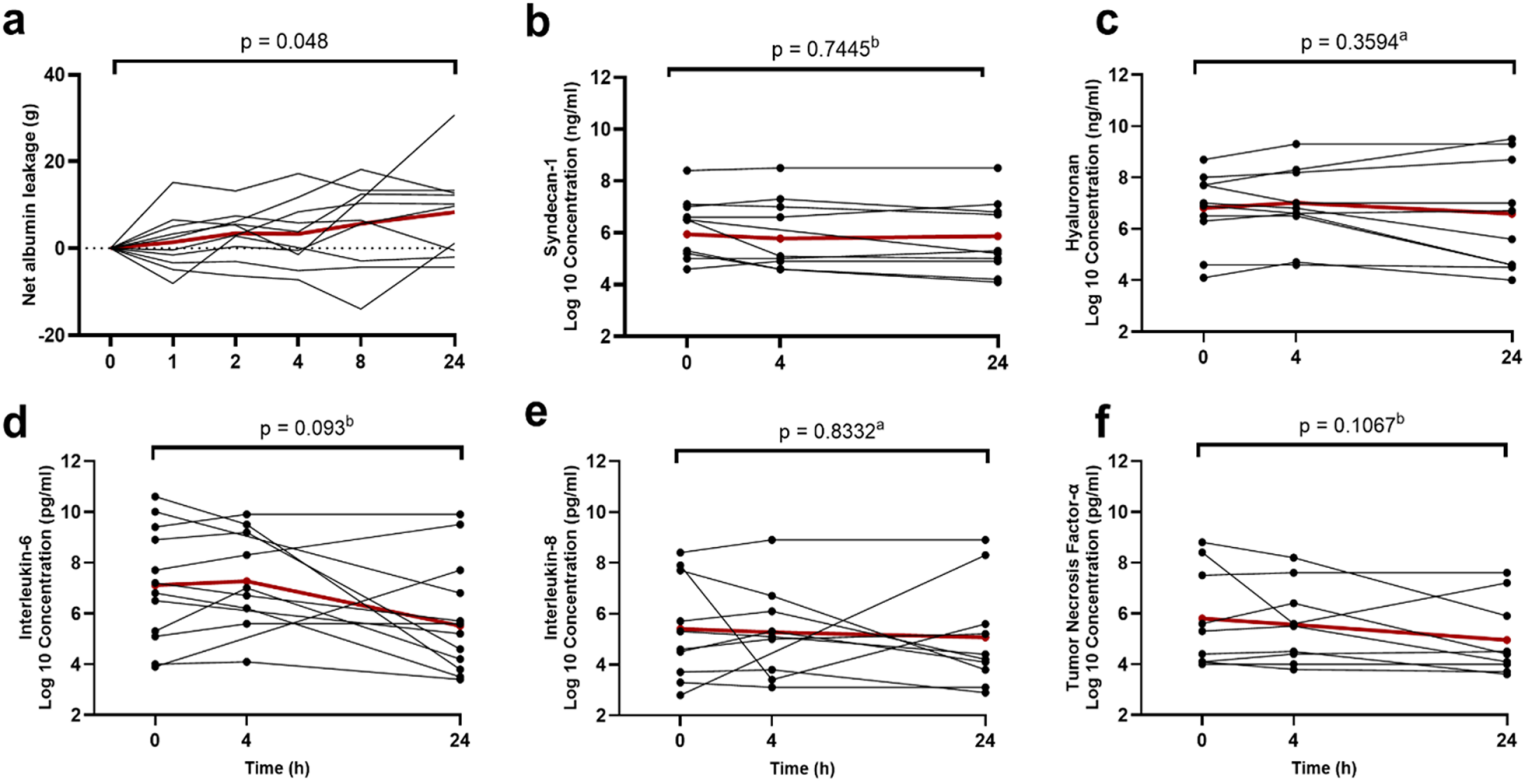
Temporal pattern of NAL **a** syndecan-1 **b**, hyaluronan **c**, interleukin-6 **d**, interleukin-8 **e**, tumor necrosis factor-α **f**. Bold red line denotes the mean in 10 patients. ^a^ repeated measures analysis of variance, ^b^ mixed effects model

NAL was 8 ± 10 g, (*p* = 0.029) at 24 h. Values are presented in Table 2, and the time courses are presented in Fig. 2. Six patients received exogenous albumin 11 g (0–70; *n* = 10). Four patients received red blood cell transfusion 0 g (0–199; *n* = 10). Plasma albumin levels decreased from 20.5 ± 5.5 to 19.6 ± 5.4 g/L (*p* = 0.53). Hemoglobin levels decreased from 89 (72–149) to 85 (64–126) g/L (*p* = 0.08). The plasma volume increased from 3.7 ± 0.6 to 4.1 ± 0.9 L (*p* = 0.08). The fluid balance was 3.7 ± 3.1 L (*p* = 0.005) and the weight gain 3.5 ± 4.4 kg (*p* = 0.03). One patient died after 13 h but was included fully in the analysis.

**Table 2:** Levels of net albumin leakage, glycocalyx shedding products, cytokines and fluid balance

	Hour 0	Hour 4	Hour 24	*p*-value	Reference ranges^1^
Net albumin leakage [g]	0	3 ± 8	8 ± 10	0.049^a^	NA
Syndecan-1 [ng/ml]	410 (140—1122)	155 (112—1208)	445 (165—840)	0.69^b^	26–439
Hyaluronan [ng/ml]	1081 (418—3294)	815 (527—4234)	524 (95—6932)	0.22ä^a^	5–24
Interleukin-6 [pg/ml]	1114 (132—14,556)	973 (322—12,114)	188 (40—1214)	0.25^b^	1–2
Interleukin-8 [pg/ml]	155 (38—2432)	171 (37—613)	73 41—1238)	0.80^b^	4–10
Tumor necrosis factor-α [pg/ml]	203 (60—3216)	249 (69—1334)	82 (51—615)	0.20^b^	12–32
Fluid balance [L]	0	1.3 ± 0.8	3.7 ± 3.2	0.0058^a^	NA

Values are presented as median (range) or mean ± standard deviation, as appropriate. ^1^ based on analysis of samples from 35 healthy volunteers (24 females, 11 males), aged 39.5 (22–50) years, with a height of 1.68 (1.55–1.90) m, and a body weight of 87.2 (69.6–138.8) kg(22). ^a^Anova with the Greenhouse–Geisser correction, ^b^Mixed effects model. NA = Not applicable

### Glycocalyx shedding products of and cytokines

Levels of syndecan-1 and hyaluronan, IL-6, IL-8 and TNF-α were all high compared to reference ranges but there was no significant change over time in any of them (Table 2; Fig. 2).

### NAL: correlations with cytokines, glycocalyx shedding and fluid balance

**Fig. 3:**
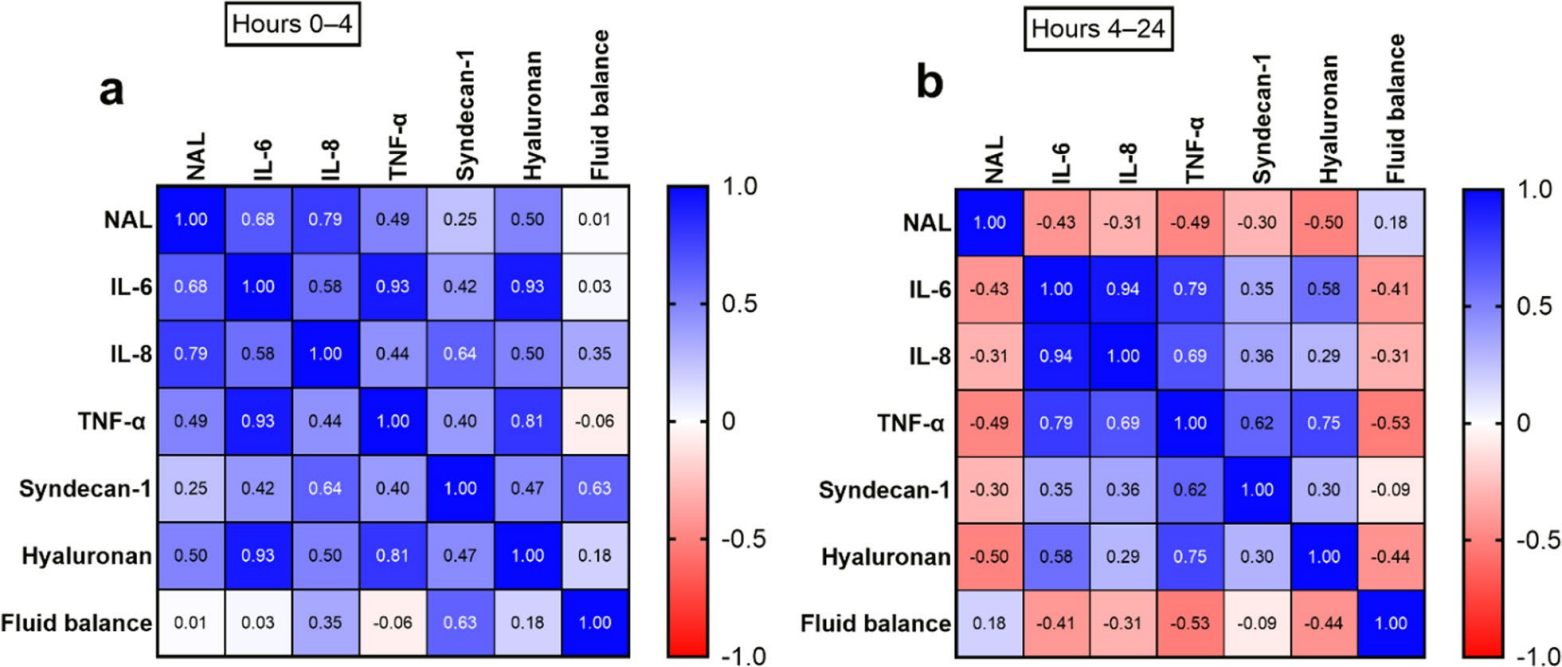
Correlation matrix (Pearson’s coefficient) in 9 patients between net albumin leakage (NAL) and the area under the curve of interleukin-6 (IL-6), interleukin-8 (IL-8), tumor necrosis factor-α (TNF-α), syndecan-1, hyaluronan, and fluid balance levels in the time period 0–4 h in the ICU **a** and 4–24 h in the ICU **b**. Data from one patient are missing due to incomplete sampling. To achieve a *p*-value of < 0.05, the Pearson correlation coefficient needs to be ≥ 0.66

At the time interval 0–4 h, there were significant correlations between NAL and IL-6 (r_s_ = 0.68, *p* = 0.045) and IL-8 (r_s_ = 0.79, *p* = 0.012) (Fig. 3a). Furthermore, IL-6 correlated to TNF-α (r_s_ = 0.93, *p* = 0.0002) and hyaluronan (r_s_ = 0.93, *p* = 0.0002). Subsequently hyaluronan correlated to TNF-α (r_s_ = 0.81, *p* = 0.0008).

In the time interval 4–24 h, NAL did not correlate with any of the shedding products or cytokines (Fig. 3b). There were no correlations with fluid balance. However, IL-6 correlated with TNF-α (r_s_ = 0.79, *p* = 0.011) and IL-8 (r_s_ = 0.94, *p* = 0.0001). Additionally, IL-8 correlated with TNF-α (r_s_ = 0.69, *p* = 0.04). Finally, TNF-α correlated with hyaluronan (r_s_ = 0.75, *p* = 0.02).

### Factors contributing to NAL, post hoc and subgroup analysis

**Fig. 4:**
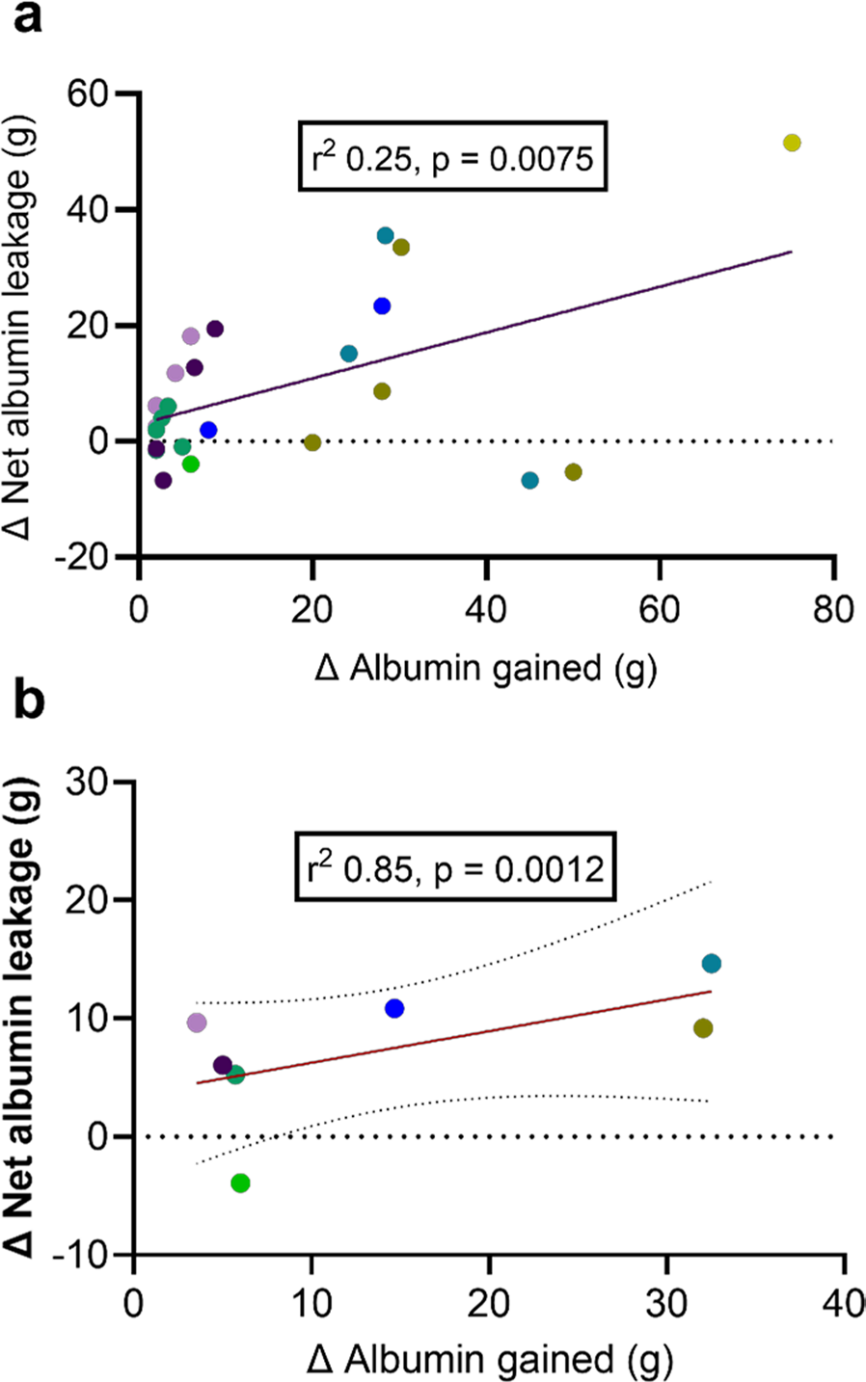
**a** Temporal pattern of the Δ albumin gained versus Δ NAL in 8 patients at 27 different timepoints in the first 82 h of ICU stay. Each patient is represented with its own color and each dot represents the amount of albumin gained by a patient (x-axis) and the subsequent corresponding change in NAL (Δ NAL) between 4 (1–46) hours later (Y = 0,3859*X + 3,567, r^2^ = 0.25, *p* = 0.0075) **b** the mean change in Δ albumin versus mean Δ NAL in the same patients, represented by the same color as in **a** (Y = 0.6180*X—0.5863, r.^2^ = 0.85, *p* = 0.0012)

The plasma albumin concentration at the time of ICU enrollment was not significantly associated with NAL (r_s_ = 0.48, *p* = 0.16). Similarly, the total amount of albumin gained over 24 h did not correlate with NAL at 24 h (r_s_ = 0.50, *p* = 0.13). However, we analyzed the relationship between the change in albumin gained (∆ Albumin gained) and the change in NAL (∆ NAL) at the next available time point. This analysis revealed a significant correlation (r^2^ = 0.25, *p* = 0.0075), as shown in Fig. 4a. When examining the mean ∆ NAL versus the mean ∆ Albumin gained for each patient, the strength of the correlation increased (r^2^ = 0.85, *p* = 0.0012), as shown in Fig. 4b. In a subgroup analysis of patients with hematologic or metastatic cancer (n = 5), the median net albumin leakage was 0 g (range: − 4 to 31 g, *p* = 0.81), with a mean of 7 g.

## Discussion

### Study overview and primary findings

In this prospective, single-center pilot study, we assessed NAL in 10 ICU patients with suspected sepsis using mass balance calculations over 24 h. The NAL at 24 h was 8 ± 10 g. We explored correlations between NAL, glycocalyx shedding products, and inflammation markers. Initially. Within the first four hours post-enrollment, significant correlations emerged between NAL and the pro-inflammatory cytokines IL-6 and IL-8, but not with glycocalyx shedding products or fluid balance. From hours 4 to 24, correlations were observed between TNF-α and hyaluronan, but these did not extend to NAL. We also noted that albumin administration appeared to increase NAL, and a signal indicating that a higher serum albumin level at ICU admission correlated with increased NAL.

### Interpretations

Surprisingly, our cohort of severely ill patients had a lower NAL, 8 ± 10 g, compared to elective patients undergoing major surgery 24 ± 17 g [11]. In the surgical patients, measurements started before any other intervention and before the physiological changes associated with major surgery. Thus, we had an appropriate time point zero. This is in contrast with septic patients, where the time of onset of physiological perturbations might occur days before hospital admission, and then, in our study, further delayed until study start in the ICU. We speculate that most of the NAL might have happened before ICU admission, and that some of our patients already were in a new steady state where the increased TER was compensated by a similarly increased lymphatic return, and that this might explain our low NAL values. However, it is equally possible that the trauma and inflammation caused by major surgery causes a more acute and pronounced NAL.

TER has previously been shown to be increased in patients with stable disease such as hypertension [24] or cancer [10]. If patients are in a steady state, this means that the size of lymphatic return of albumin must be identical to albumin leakage through TER, and that the NAL value must be zero. This demonstrates that a TER value as such cannot be interpreted as an index of albumin net leakage. To our knowledge, the only published value of TER in septic ICU patients is 13.4%/h, like in stable cancer patients in the same study, but more than twice that of healthy volunteers [10]. If cancer patients can balance such a high TER with an increased lymphatic return, we cannot see why this would not be possible also for septic patients after an initial albumin leakage.

Our cohort had a net plus fluid balance and weight gain after 24 h. In the absence of correlations with NAL, consistent with the findings of Norberg [11], this suggests that albumin might potentially mobilize from the interstitium back to circulation more rapidly than fluid.

Shedding products such as syndecan-1 and hyaluronan were elevated, as previously reported in critically ill patients [18], but no correlations to NAL were observed. We are unaware of any studies using radioactive tracers to estimate TER and link it to shedding products, which would arguably be more intuitive than linking it to NAL. However, when Hahn et al. administered albumin to burn patients and used mass balance calculations to estimate a TER equivalent, they found no correlations between syndecan-1 and albumin leakage [25].

Early correlations between NAL and IL-6 and IL-8, dissipating after four hours, highlight the transient nature of inflammatory responses in sepsis. However, we must underscore that these findings are only hypothesis-generating acknowledging the risk of spurious correlations due to multiple testing.

In our post hoc analysis we correlated albumin levels with NAL and found a positive value but that did not reach statistical significance. With only ten patients it is likely that our study is underpowered to detect a true correlation needing an r^2^ ≥ 0.39 to be significant and thus this phenomenon may warrant further analysis in a larger study. The theoretical reasoning for this analysis was that even though the revised Starling equation [26] de-emphasizes the role of the colloid osmotic pressure difference between the intravascular and interstitial spaces, inflammation-induced expression of large pores [27] might demask the large concentration gradient between the intravascular space and the interstitium, thereby accelerating leakage. High albumin levels coinciding with a high NAL may then also indicate an early stage of disease where a new steady state between TER and lymphatic return has not been reached. Conversely, an association of low albumin levels with low NAL could suggest a later stage in the disease course where higher concentration of interstitial albumin, which might enhance the return of albumin per volume of lymphatic flow.

Finally, a post hoc regression analysis revealed that any form of albumin supplementation was associated with an increased NAL. We attribute this finding to the fact that all exogenous drugs will eventually be distributed throughout the entire pool. We consider it a strength of our method that it can detect this anticipated outcome.

### Strengths and limitations

This study provides new data on albumin kinetics in a very sick cohort with sepsis, describing the net effects of capillary leakage counterbalanced by lymphatic return. The method is easy to use, minimally invasive and allows NAL estimation within a few hours depending on the laboratory availability. The correlations found can be used to generate hypothesis for future studies.

Our study has limitations. The use of mass balance calculations for NAL in septic patients is novel and lacks a defined "time zero" compared to surgical cohorts. Enrolment occurred within 12 h of ICU admission, and patients could be recruited from either the ward or emergency room. Rapid plasma volume changes can occur quickly and likely complicated interpretations of albumin homeostasis preceding enrolment [22, 28]. Additionally, low plasma albumin levels suggest a more prolonged septic course, and our decision to initiate blood sampling only upon ICU admission may have missed earlier dynamic changes. Surgical interventions during the study also introduced potential confounders, such as unaccounted occult bleeding. Furthermore, other factors influencing mass balance calculations, like non-bleeding-related anemia, third space losses that were not drained and therefore not sampled, and altered albumin metabolism, were not fully explored. Although Norberg [11] published case report simulations indicating moderate influence on NAL by different start estimates for blood volume, this may be a larger problem in critical care patients. The standard error in anthropometric calculation of blood volume in healthy individuals has been reported as 0.4 L [21] or 12% [29]. Different formulas have also been suggested for patients that deviate a lot from ideal body weight [30]. To our knowledge, there is no validated formula for blood volume estimates in critical illness, and a development here would likely improve the quality of mass balance calculations. Our septic cohort was mainly within the normal range of BMI but might have been hypovolemic upon recruitment. Because a lower start estimate of blood volume results in a slightly larger NAL, this might have contributed to an underestimation of NAL. The measurement of TER and estimations on the lymphatic return would have provided additional valuable insights, but both faced logistical challenges that surpassed the scope of this study. Finally, we cannot fully determine how the glycocalyx influences mass balance calculations. Hypothetically albumin could be recruited back to the circulation from the shedding glycocalyx, potentially leading to an underestimation of NAL. Additionally, if the glycocalyx 'stands up' [31] or is rebuilt, this may reduce the distribution volume of erythrocytes possibly increasing hemoglobin levels while albumin levels are unaffected, and therefore decreasing NAL. Conversely, if it lies flat or sheds, the opposite effect could occur.

## Conclusion

In a 24-h period, our study observed a NAL of 8 ± 10 g from the circulation, presumably into the interstitium, in 10 patients with suspected sepsis.

There was a weak correlation between NAL and pro-inflammatory cytokines during the first 4 h of ICU stay. However, no correlation was found between NAL and either fluid balance or glycocalyx shedding products.

The administration of albumin infusions appeared to increase the NAL.

## Supplementary Information

Additional file1 (DOCX 23 KB)

## Data Availability

The datasets used and analyzed during the current study are available from the corresponding author on reasonable request.

## Abbreviations

NAL: Net albumin leakage
IL-6: Interleukin-6
IL-8: Interleukin-8
IL-10: Interleukin-10
TNF-α: Tumor necrosis factor-α
BMI: Body mass index
CRP: C-reactive protein
TER: Transcapillary escape rate
IAM: Intravascular albumin mass
MHb: Intravascular hemoglobin mass
FFP: Fresh frozen plasma
Hct: Hematocrit
ELISA: Enzyme-linked immunosorbent assay
