# Supplementary material for: Net albumin leakage in patients in the ICU with suspected sepsis. A prospective analysis using mass balance calculations
Source: Crit Care. 2025 Mar 8;29:106. doi: 10.1186/s13054-025-05323-9 (PMC11890723; doi:10.1186/s13054-025-05323-9)
Supplement: Supplementary file 1 — Additional file1 (DOCX 23 KB) [file 13054_2025_5323_MOESM1_ESM.docx]

# SUPPLEMENTARY APPENDIX: NET ALBUMIN LEAKAGE IN PATIENTS IN THE ICU WITH SUSPECTED SEPSIS. A PROSPECTIVE ANALYSIS USING MASS BALANCE CALCULATIONS

Dag Seldén

Perioperative Medicine and Intensive Care, Karolinska University Hospital, Huddinge

Department of Clinical Science Intervention and Technology (CLINTEC), Karolinska Institutet, Hälsovägen, Stockholm, Sweden

dag.selden@ki.se

Nicolas Tardif

Department of Clinical Science Intervention and Technology (CLINTEC), Karolinska Institutet, Hälsovägen, Stockholm, Sweden

nicolas.tardif@ki.se

Jan Wernerman

Perioperative Medicine and Intensive Care, Karolinska University Hospital, Huddinge

Department of Clinical Science Intervention and Technology (CLINTEC), Karolinska Institutet, Hälsovägen, Stockholm, Sweden

jan.wernerman@ki.se

Olav Rooyackers

Perioperative Medicine and Intensive Care, Karolinska University Hospital, Huddinge

Department of Clinical Science Intervention and Technology (CLINTEC), Karolinska Institutet, Hälsovägen, Stockholm, Sweden

olav.rooyackers@ki.se

Åke Norberg

Perioperative Medicine and Intensive Care, Karolinska University Hospital, Huddinge

Department of Clinical Science Intervention and Technology (CLINTEC), Karolinska Institutet, Hälsovägen, Stockholm, Sweden

ake.norberg@ki.se

## Calculations of Mass Balance

The following calculations of mass balance are reproduced from Norberg et al. (11), as they form the methodological basis for this study.

Baseline blood volume (BV) was calculated anthropometrically using the following formulas by Nadler (21):

BV = 0.6041 + 0.03219 x body weight (kg) + 0.3669 x (body length (m))^3^ (Males)

BV = 0.1833 + 0.03308 x body weight (kg) + 0.3561 x (body length (m))^3^ (Females)

Using these BVs plasma volume (PV) was derived by:

PV = (1 – B-Hct × 0.91) × BV. (1)

The f-ratio 0.91 represents the ratio between total body hematocrit and large vessel B-Hct. The intravascular hemoglobin mass (MHb) is then:

MHb = BV × B-Hb (2)

By combining (1) and (2) we achieve:

PV = (1 – B-Hct × 0.91) × MHb / B-Hb (3)

Intravascular albumin mass (IAM) can be similarly calculated by:

IAM = PV × P-alb (4)

Equations 1–4 are valid at all time points. When considering consecutive time points (*n* + 1 versus *n*) in the predefined protocol, MHb at time *n* + 1 can be calculated from the value at time n and measured loss and gain of hemoglobin in that time interval according to:

MHb_n_+1 = MHb_n_ – bleeding volume × mean B-Hb + transfusion of Hb (5)

When this MHb*_n_*+1 from equation (5) is inserted into equation (3) together with B-Hb*_n_*_+1_ and B-Hct*_n_*_+1_, it is possible to calculate PV*_n_*_+1_. This value can be inserted into equation (4) together with P-alb*_n_*_+1_ generating IAM*_n_*_+1_, representing albumin mass at time point *n* + 1 related only to B-Hb and B-Hct and measured loss and gain of hemoglobin. IAM' represents another way to assess mass balance of albumin directly by considering losses and gains of albumin over time. The apostrophe denotes that these IAM' values are obtained differently. Gains are estimated from albumin content in plasma transfusions, platelet transfusions and albumin infusions, losses from measurement of albumin in drains, urinary catheters or from estimated bleeding in suction bottles and sponges according to:

Albumin loss = (bleeding volume × mean P-alb × (1−mean B-Hct)) + (urinary albumin concentration × urine volume) + (drain albumin concentration × drain fluid volume) (6)

The net difference between these two measures over time is presented in this paper as net albumin leakage, supposedly to the interstitium:

NAL = IAM' – IAM (7)

Finally, the fractional plasma volume dilution at time n (fPVdil*_n_*) is then related to the baseline plasma volume PV_0 according to:

fPVdil*_n_* = PV*_n_* / PV_0_  (8)

Similar fPVdil calculations serve as in-data in volume kinetic modelling (30).
